# Radiomics nomogram based on multi-parametric magnetic resonance imaging for predicting early recurrence in small hepatocellular carcinoma after radiofrequency ablation

**DOI:** 10.3389/fonc.2022.1013770

**Published:** 2022-11-10

**Authors:** Xiaojuan Zhang, Chuandong Wang, Dan Zheng, Yuting Liao, Xiaoyang Wang, Zhifeng Huang, Qun Zhong

**Affiliations:** ^1^ Department of Radiology, Fujian Medical University Xiamen Humanity Hospital, Xiamen, China; ^2^ Fuzong Clinical Medical College of Fujian Medical University, Fuzhou, China; ^3^ Department of Thyroid and Breast Surgery, Fujian Medical University Xiamen Humanity Hospital, Xiamen, China; ^4^ Shengli Clinical Medical College of Fujian Medical University, Fuzhou, China; ^5^ Department of Radiology, 900th Hospital of Joint Logistics Support Force, Fuzhou, China; ^6^ Institute of Precision Medicine, GE Healthcare, Shanghai, China; ^7^ Department of Radiology, The Affiliated People’s Hospital of Fujian University of Traditional Chinese Medicine, Fuzhou, China

**Keywords:** small hepatocellular carcinoma, radiofrequency ablation, early recurrence, magnetic resonance imaging, radiomics, nomogram

## Abstract

**Background:**

There are few studies on the application of radiomics in the risk prediction of early recurrence (ER) after radiofrequency ablation (RFA). This study evaluated the value of a multi-parametric magnetic resonance imaging (MRI, mpMRI)-based radiomics nomogram in predicting ER of small hepatocellular carcinoma (HCC) after RFA.

**Materials and methods:**

A retrospective analysis was performed on 90 patients with small HCC who were treated with RFA. Patients were divided into two groups according to recurrence within 2 years: the ER group (n=38) and the non-ER group (n=52). Preoperative T1WI, T2WI, and contrast-enhanced MRI (CE-MRI) were used for radiomic analysis. Tumor segmentation was performed on the images and applied to extract 1316 radiomics features. The most predictive features were selected using analysis of variance + Mann–Whitney, Spearman’s rank correlation test, random forest (importance), and least absolute shrinkage and selection operator analysis. Radiomics models based on each sequence or combined sequences were established using logistic regression analysis. A predictive nomogram was constructed based on the radiomics score (rad-score) and clinical predictors. The predictive efficiency of the nomogram was evaluated using the area under the receiver operating characteristic curve (AUC). Decision curve analysis (DCA) was used to evaluate the clinical efficacy of the nomogram.

**Results:**

The radiomics model mpMRI, which is based on T1WI, T2WI, and CE-MRI sequences, showed the best predictive performance, with an AUC of 0.812 for the validation cohort. Combined with the clinical risk factors of albumin level, number of tumors, and rad-score of mpMRI, the AUC of the preoperative predictive nomogram in the training and validation cohorts were 0.869 and 0.812, respectively. DCA demonstrated that the combined nomogram is clinically useful.

**Conclusions:**

The multi-parametric MRI-based radiomics nomogram has a high predictive value for ER of small HCC after RFA, which could be helpful for personalized risk stratification and further treatment decision-making for patients with small HCC.

## 1 Introduction

Hepatocellular carcinoma (HCC) is the most common primary malignant tumor of the liver and one of the main causes of cancer-related deaths (1, 2). With the continuous development of diagnostic equipment and technology, an increasing number of HCC can be diagnosed at early stages. According to the Barcelona Clinical Liver Cancer (BCLC) staging system, for very early stage (BCLC-0 stage) and early stage (BCLC-A stage) HCC, that is, small HCC with single or at most three cancerous nodules with a diameter ≤ 3 cm, the treatment includes surgical resection, liver transplantation, and radiofrequency ablation (RFA) (1). However, choosing the best treatment for HCC remains a complex problem. In recent years, owing to the limited use of surgical resection and liver transplantation for various reasons, such as limited liver reserve, shortage of organ donors, and high incidence and mortality of surgical complications, RFA has been recommended as the first-line treatment choice for patients with small HCC (3, 4). The overall survival rate of patients treated with RFA is similar to that of patients treated with surgical resection (5). Moreover, in the past few decades, RFA technology has made continuous progress, gradually strengthening local tumor control and reducing the occurrence of complications (6). However, during follow-up of patients treated with RFA, HCC still has a high recurrence rate, which may be as high as 70% within 5 years (7).

According to the latest clinical practice guidelines for HCC, recurrence of HCC can be divided into early recurrence (ER) (< 2 years) and late recurrence (> 2 years) (8, 9). ER is generally considered a result of occult metastasis of the primary tumor, while late recurrence is considered to be a new HCC in the context of liver cirrhosis (10–13). This is consistent with the genetic analysis of tumor recurrence; early recurrent tumors are likely to show a similar clonal origin as preoperative primary tumors, whereas late recurrent tumors often show different clonal origins (12). Meanwhile, a growing number of studies have shown that ER is mostly related to the biological characteristics of primary tumors, including tumor size and number, microvascular invasion and poor histological differentiation, as well as preoperative serum alpha-fetoprotein (AFP) and albumin (ALB) levels (10, 13–16). HCC with ER usually has a poor prognosis (17). Preoperative identification of high-risk patients with ER can guide surgical treatment, postoperative monitoring, and treatment intervention. At present, several studies (18–20) have tried to predict the prognosis of patients with HCC by preoperative conventional imaging examination, but the conditions for accurate prediction of ER treated with RFA by these methods are not yet mature and can be affected by subjective factors. Therefore, there is still a lack of objective and reliable preoperative prediction methods, with ER remaining one of the main barriers to improving the prognosis of patients.

Recently, radiomics has been widely used to evaluate tumor invasiveness and prognosis by extracting and evaluating features from medical images (21–23). Notably, HCC is a complex neoplastic lesion that develops from multi-step carcinogenesis of cirrhotic nodules, during which there are significant changes in blood supply, accompanied by multiple immature abnormal angiogenesis. Excessive proliferation of tumor cells leads to an increase in cell atypia, a change in cell membrane permeability, and metabolic processes (24). These micropathological changes lead to mixed internal components and uneven structure of the tumor, and the images show tumor heterogeneity information, such as rough texture and complex gray distribution, which can be captured by radiomics. The higher the heterogeneity of the tumor, the stronger the invasiveness of the tumor and the worse its prognosis (21, 22, 24). Some studies have predicted the ER or survival time of HCC by preoperative enhanced computed tomography or magnetic resonance imaging (MRI) radiomics models (25, 26). However, most of these studies focused on the observation of ER after surgical resection, ignoring patients treated with RFA. At present, there are few studies on the application of radiomics in the risk prediction of ER after RFA, and its feasibility is unclear. Meanwhile, MRI has the characteristics of multi-parameter, multi-sequence, and multi-directional imaging, which may contain more tumor heterogeneity information undetectable by the naked eye, and this will provide more possibilities for predicting the prognosis of small HCC before RFA. Therefore, our study aimed to explore the feasibility of multi-parametric MRI (mpMRI)-based radiomics to predict the ER of small HCC after RFA and to establish a nomogram based on the combination of radiomics score (rad-score) and clinical features to explore its value in individualizing the prediction of ER in small HCC patients.

## 2 Materials and methods

### 2.1 Patients

This was a retrospective review of all patients with HCC treated with RFA at the 900th Hospital of the Joint Logistics Support Force, Fuzhou, China, between January 2018 and January 2020. The criteria for the study were as follows: (1) confirmed by pathology or diagnosed with liver cancer in strict accordance with the Chinese Standard for diagnosis and treatment of primary liver cancer (2017 Edition) (27); (2) the tumor was single or multiple (number ≤ 3, and diameter ≤ 3 cm), and there was no vascular invasion or extrahepatic metastasis; (3) RFA was used as a first-line treatment and was examined by contrast-enhanced MRI (CE-MRI) within one month before the operation; (4) complete clinical and laboratory data; and (5) follow-up time of more than two years. Patients with poor MRI image quality or those complicated by other tumors were excluded. Finally, 90 patients were enrolled in the study, 38 of whom had ER, while the others did not. This study was reviewed and approved by the Institutional Review Board of the 900th Hospital of the Joint Logistics Support Force. The data were anonymized, and the requirement for informed consent from the patients was waived. All study procedures were performed in accordance with the Helsinki Declaration of 1964 and its later versions.

### 2.2 Clinical characteristics

Preoperative clinical data of the patients were collected in detail, including sex, age, etiology, history of liver cirrhosis, Child-Pugh grade (grade A or B), AFP, alanine aminotransferase, aspartate aminotransferase, ALB, bilirubin, tumor number, tumor size, and location.

#### 2.2.1 MRI procedure

All patients were examined by 1.5T or 3.0T MRI scanners (GE SignaHDx or Discovery 750; Siemens Magnetom Trio Tim or Skyra). The scanning sequences included T1WI, T2WI, multi-phase enhanced T1WI, and diffusion-weighted imaging (DWI). The corresponding parameters of different sequences are as follows: (1) T1WI (Repetition time [TR] 4 ms, echo time [TE] 2 ms) uses layer thickness 5 mm, layer spacing 2.5 mm, matrix 288 × 192, excitation times 1; (2) T2WI (TR 8500 ms, TE 90 ms) layer thickness 5 mm, layer spacing 5 mm, matrix 320 × 224, excitation times 1; (3) multi-phase enhanced T1WI sequence (TR 4 ms, TE 2 ms; field of vision 360 × 400 mm; layer thickness 5 mm; layer spacing 2.5 mm, matrix 288 × 192), wherein three phase enhanced scans were performed, including the arterial phase (AP), portal venous phase (PVP), and delayed phase (DP). The contrast agent was Gadobenate Dimeglumine (MultiHance; Bracco, Shanghai, China) and was injected at a patient weight-dependent dose of 0.2 ml/kg and injection rate of 2.0 ml/sec through the median cubital vein. (4) DWI: visual field 400 × 343 mm; matrix 116 × 97; TR 2500 ms, TE 64 ms; slice thickness 7 mm; interlayer spacing 1 mm; b=800 sec/mm^2^.

### 2.3 Follow-up

Complete ablation confirmed with a dynamic CT or MRI scan was performed 1 month post‐RFA. Patients were followed up for at least 2 years after RFA every 3–6 months. Recurrence was monitored using AFP level, liver function, computed tomography, or MRI. ER is defined as a new cancerous focus with typical imaging features of the liver or other organs within 2 years of RFA treatment.

### 2.4 Image preprocessing and tumor segmentation

T1WI, T2WI, and CE-MR (AP, PVP, and DP) images were converted to Medical Digital Imaging and Communication format. Image preprocessing was performed using AK (Artificial Intelligence Kit, GE Healthcare). Each image is resampled by 1.0 mm × 1.0 mm × 1.0 mm, and then z-Score transform is performed to transform the image intensity into standard normal distribution, wherein mean is 0 and standard deviation is 1.

**Figure 1:**
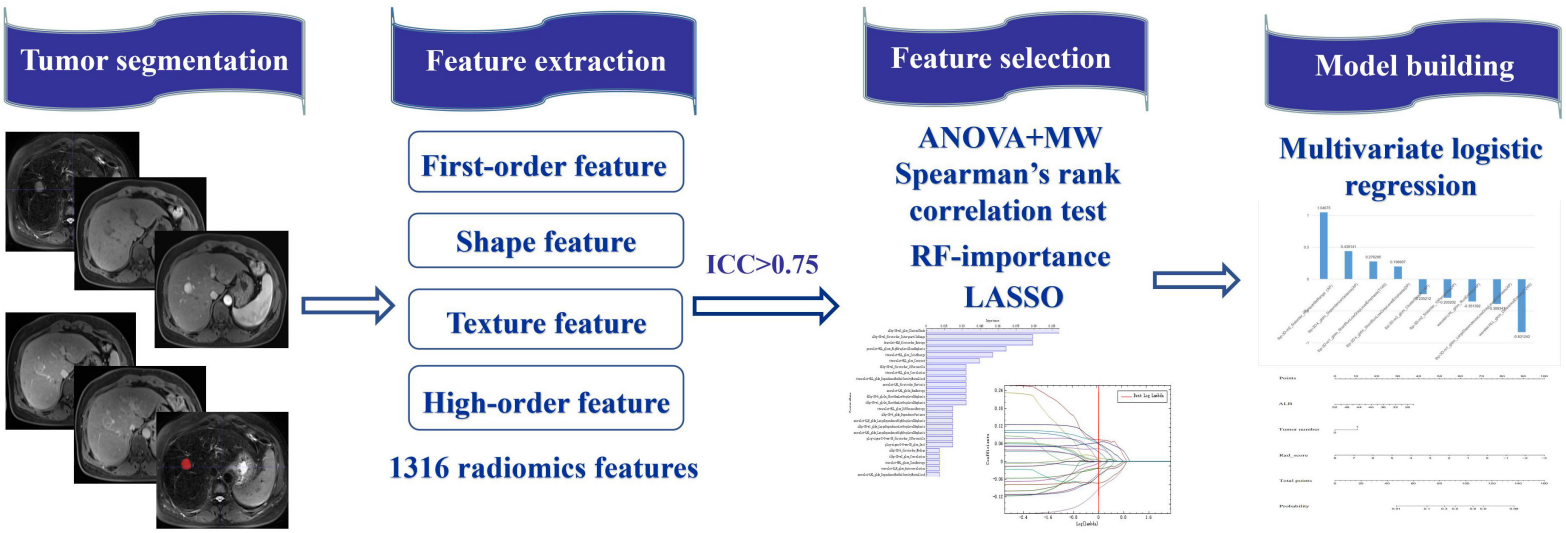
The workflow of radiomics in the current study. Tumor 2D segmentation was performed on mpMRI and applied to extract 1316 radiomics features, including first-order feature, shape feature, texture feature and higher-order feature. After interobserver agreement analysis, the most predictive radiomics features were selected *via* the Anova+MW, Spearman’s rank correlation test, RF-importance, and LASSO analysis. The radiomics model was established by multivariate logistic regression analysis. The predictive nomogram was constructed based on the radiomics score and clinical predictors.

Tumor segmentation was performed by two physicians with rich experience in radiological diagnosis, wherein physician A used the open-source software ITK-SNAP (www.itk-snap.org) to draw the regions of interest (ROI) on the maximum cross-section of the tumor. DP- or T2-weighted images were used as references (**Figure 1**).

### 2.5 Radiomics feature extraction and intra-group correlation coefficient (ICC) evaluation

A total of 1316 radiomics features were extracted from each tumor segment, including first-order histogram features (intensity distribution of the internal ROI), shape features (morphological features of the lesions), texture features (heterogeneity through the relative spatial position of voxels, including gray-level co-occurrence matrix, gray-level run length matrix, gray-level size zone matrix, neighboring gray tone difference matrix, gray-level dependence matrix, and local binary pattern), high-order features (adding filters or high-order image description indicators, including Laplacian of Gaussian), and wavelet features (22).

ICC was used to evaluate the repeatability between the observers of radiomics feature extraction. When ICC was > 0.75, feature extraction has good consistency. Fifteen images were randomly selected and segmented by physician B at an interval of two weeks. According to the above method, the features were extracted and evaluated using ICC. The features with ICC < 0.75 were removed to ensure the stability and repeatability of feature extraction.

### 2.6 Feature selection and radiomics model construction

All patients were randomly divided into training and validation cohorts at a ratio of 7:3. Feature selection and model construction of the training and validation cohorts were used to verify the generalization of the model.

To select the most distinctive radiomics features between the ER and non-ER groups, the selected features were screened using the AK native algorithm. The Analysis of Variance + Mann–Whitney algorithm was initially used to exclude features with no significant differences (variance difference is 0), and Spearman’s rank correlation test was used to eliminate redundant features (correlation coefficient was greater than 0.90). Then, using the random forest importance algorithm (28), we further selected the 25 features with the highest importance. Finally, features that were highly related to ER were extracted by the least absolute shrinkage and selection operator algorithm (29).

Then, a radiomics model in each MRI sequence was constructed using logistic regression analysis to predict ER, and models based on the combination of different MRI sequences were established, including CE-MRI (AP+PVP+DP) and mpMRI (T1WI+T2WI+AP+PVP+DP). The rad-score was calculated according to the selected features and their corresponding coefficients using the formula: rad-score = constant + coefficients × features. The area under the receiver operating characteristic (ROC) curve (AUC) of each model in the validation cohort was compared to evaluate the model with the best predictive performance.

### 2.7 Combined nomogram model building

The combined model was established using the rad-score of the radiomics model with the best prediction performance and the highly related clinical indicators of ER by logistic regression analysis, which was presented in the form of a nomogram. Accuracy of quantitative prediction was evaluated using the AUC. The consistency between the predicted results and the actual results was evaluated using a calibration curve, and its clinical effectiveness was evaluated using a decision curve analysis (DCA).

### 2.8 Statistical analysis

Statistical analysis was performed using the SPSS 26.0 software and R software (3.6.0, http://www.r-project.org). *P* < 0.05 indicated a statistically significant difference.

Clinical data were analyzed using the SPSS software. The chi-square test was used for classified variable analysis, t-test for continuous variables of normal distribution, and Mann–Whitney U test for abnormal or unknown distribution. Univariate and multivariate logistic analyses were performed to screen for clinical indicators with a high correlation with ER.

The R software was used to establish and evaluate the nomogram. The software packages of “car,” “rms,” “pROC,” and “DecisionCurve” were used to analyze the nomogram, ROC curve, calibration curve, and DCA.

## 3 Results

### 3.1 Patient characteristics

The statistical analysis results for the baseline data of the ER and non-ER groups are shown in **Table 1**. There were significant differences in age (*P*=0.036), preoperative ALB level (*P*=0.015), and tumor number (*P*=0.010). However, other baseline characteristics were not significantly different between the two groups.

**Table 1:** The baseline data of patients with small liver cancer in ER cohort and NER cohort.

	ER group (n = 38)	NER group (n = 52)	*P* value
Age(Y)	59.29±8.71	54.29±12.40	0.036*
Gender			0.225
male female	32 (84.2) 6 (15.8)	41 (78.8) 11 (21.2)	
TBIL(μmol/L)	18.31±10.31	20.22±23.32	0.637
ALB(U/L)	38.01±5.26	41.04±6.06	0.015*
ALT(U/L)	57.62±117.03	52.07±57.71	0.767
AST(U/L)	50.49±63.26	52.57±59.43	0.874
AFP(U/L)	114.40±252.34	90.66±174.13	0.599
HBsAg	35 (92.1)	41 (78.8)	0.085
Child—Pugh			0.157
A B	28 (73.6) 10 (26.4)	46 (88.5) 6 (11.5)	
Liver cirrhosis	34 (89.5)	39 (75.0)	0.106
Tumor size(cm)	1.98±0.70	1.73±0.70	0.095
Tumor number			0.010*
≥2 1	11 (28.9) 27 (71.1)	4 (7.6) 48 (92.3)	
Tumor location			0.066
right lobe left lobe	30 (78.9) 8 (21.1)	48 (92.3) 4 (7.6)	

*P < 0.05 indicates a significant difference.

### 3.2 Correlation between clinical factors and occurrence of ER in patients

The results of the univariate and multivariate logistic regression analyses of the ER and non-ER groups are shown in **Table 2**. Univariate logistic regression analysis preliminarily screened all clinical features and found that age (*P*=0.040; hazard ratio [HR]=1.043; 95% confidence interval [CI]:1.002–1.087), ALB level (*P=*0.018; HR=0.913; 95% CI:0.846–0.985), and number of tumors (*P*=0.012; HR=4.889; 95% CI:1.418–16.885) were variables with *P* < 0.05. Multivariate logistic regression analysis further confirmed that the ALB level (*P*=0.037; HR=0.919; 95% CI:0.850–0.995) and number of tumors (*P*=0.041; HR=3.829; 95% CI:1.058–13.851) were independent predictors of ER for small HCC.

**Table 2:** Univariate and multivariate logistic regression analysis of risk factors for ER after RFA.

Variable	Univariate Analysis		Multivariate Analysis	
	HR	*P* value	HR	*P* value
Age(Y)	0.392(0.116,1.329)	0.133		
Gender	1.043(1.002,1.087)	0.040	1.041(0.997,1.041)	0.069
TBIL	0.994(0.970,1.019)	0.637		
ALB	0.913(0.846,0.985)	0.018	0.919(0.850,0.995)	0.037*
ALT	1.001(0.996,1.006)	0.766		
AST	0.999(0.992,1.006)	0.872		
AFP	1.001(0.999,1.003)	0.597		
HBsAg	3.500(0.913,13.421)	0.068		
Child-Pugh	2.379(0.767,7.386)	0.134		
Liver cirrhosis	2.833(0.844,9.514)	0.092		
Tumor size	1.682(0.911,3.106)	0.096		
Tumor number	4.889(1.418,16.885)	0.012	3.829(1.058,13.851)	0.041*
Tumor location	3.200(0.886,11.555)	0.076		

*P < 0.05 indicates a correlation between ER and factor.

### 3.3 Feature selection and establishment of image group model

After repeatability analysis among observers, the remaining characteristics of the T1WI, T2WI, AP, PVP, and DP sequences were 1064, 1148, 1156, 1017, and 946, respectively (ICC ranges: 0.751–1.000, 0.750–1.000, 0.750–0.999, 0.750–1.000, and 0.750–1.000, respectively).

**Figure 2:**
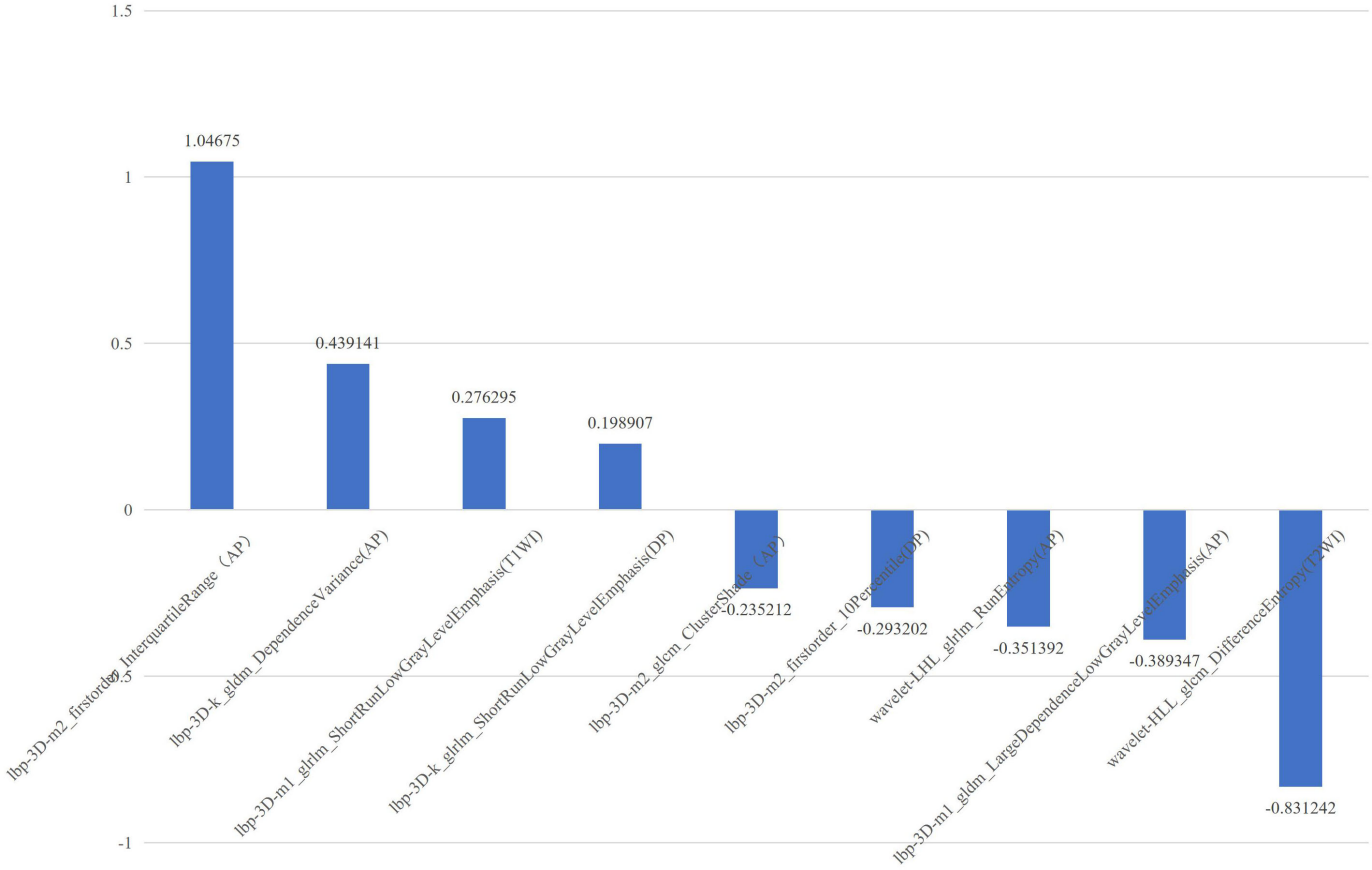
The selected features with correlation coefficients of mpMRI radiomics model. The x-axis represents radiomics features, with their coefficients in the multivariate logistic regression analysis plotted on the y-axis.

T1WI, T2WI, AP, PVP, DP, and AP radiomics models retained 8, 9, 10, 10, and 8 non-zero coefficient features, respectively, while the combined CE-MRI and mpMRI models both retained nine non-zero coefficient features. The predictive performance of the radiomics model in the validation cohort is shown in **Table 3**. Among the five models based on a single MRI sequence, the AP model performed better, with an AUC of 0.816 in the training cohort and 0.781 in the validation cohort, with an accuracy of 0.750, specificity of 0.687, and sensitivity of 0.833. The predictive performance of the combined model was better than that of the single-sequence model, and the mpMRI model (**Figure 2**) showed the best predictive performance in all models with an AUC of 0.822 in the training cohort and 0.812 in the validation cohort, with an accuracy of 0.821, specificity of 0.875, and sensitivity of 0.750.

**Table 3:** Prediction performance of each radiomics model in validation cohorts.

	T1WI	T2WI	AP	PVP	DP	CE-MRI	mpMRI
AUC	0.729	0.776	0.781	0.760	0.739	0.792	0.812
Accuracy	0.714	0.714	0.750	0.785	0.750	0.750	0.821
Specificity	0.750	0.750	0.687	0.812	0.812	0.750	0.875
Sensitivity	0.667	0.667	0.833	0.750	0.667	0.750	0.750

CE-MRI: AP+PVP+DP;mpMRI: T1WI+T2WI+AP+PVP+DP.

### 3.4 ER predictive nomogram construction

**Figure 3:**
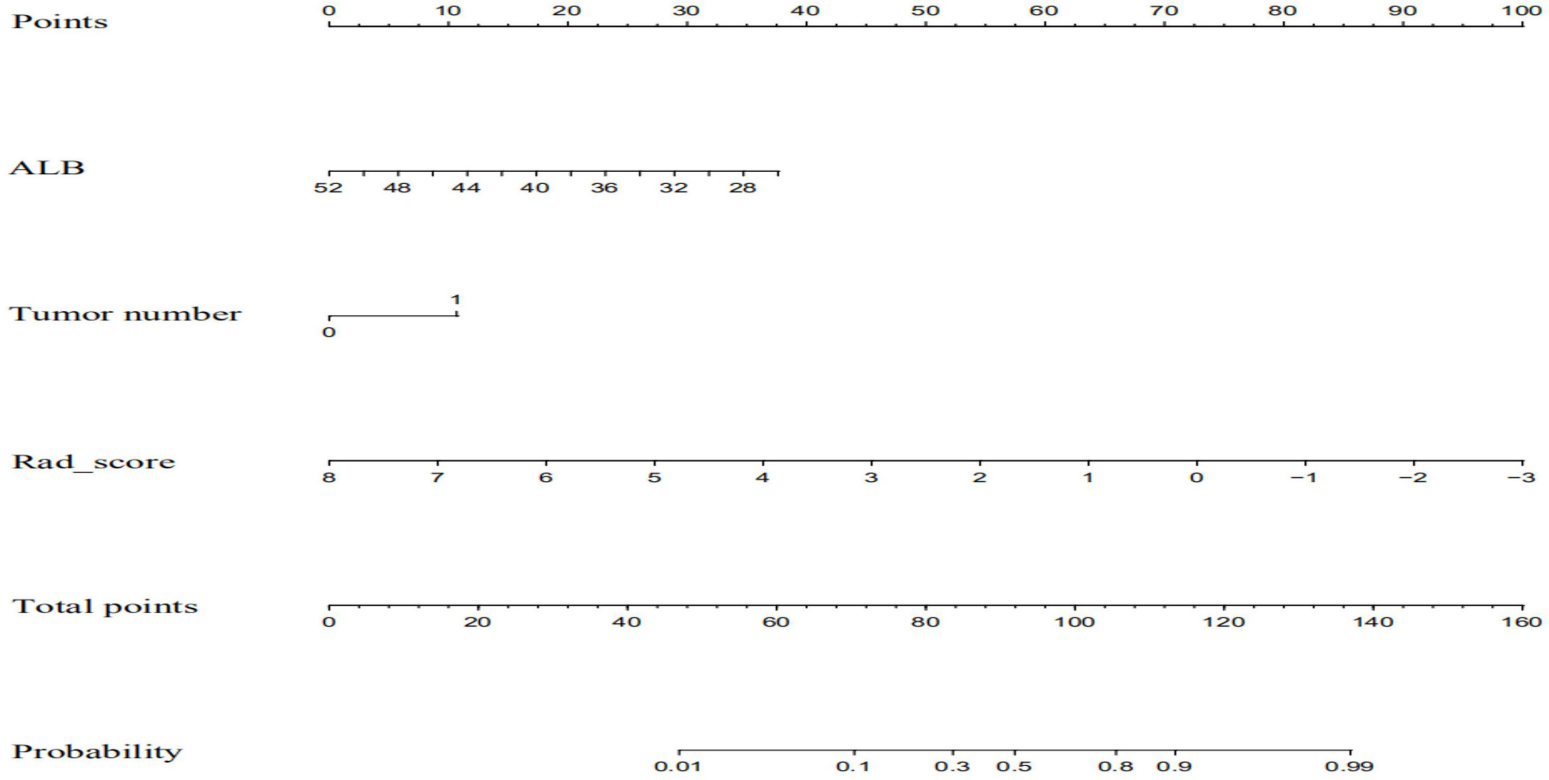
The combined nomogram incorporated ALB, tumor number, and the radiomics score. The nomogram is valued to obtain the probability of ER by adding up the points identified on the points scale for each variable albumin.

**Figure 4:**
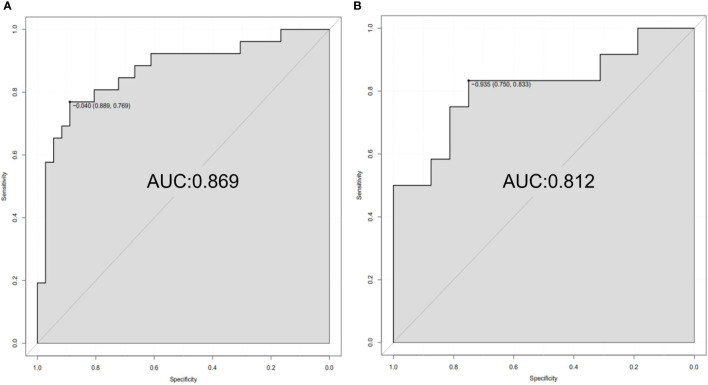
ROC curves of the combined nomogram in training **(A)** and Validation Cohorts **(B)**.

**Figure 5:**
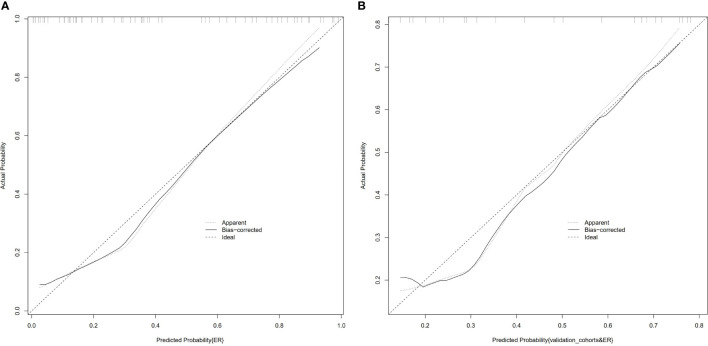
Calibration curves of the combined nomogram in the training cohort **(A)** and the validation cohort **(B)**. The vertical axis displays the actual results. The horizontal axis represents the probability of prediction. The slash dotted line represents the reference line and represents the “ideal” prediction. The solid line represents the performance of the nomogram. If the solid line is closer to the diagonal dashed line, it means that the prediction is better.

**Figure 6:**
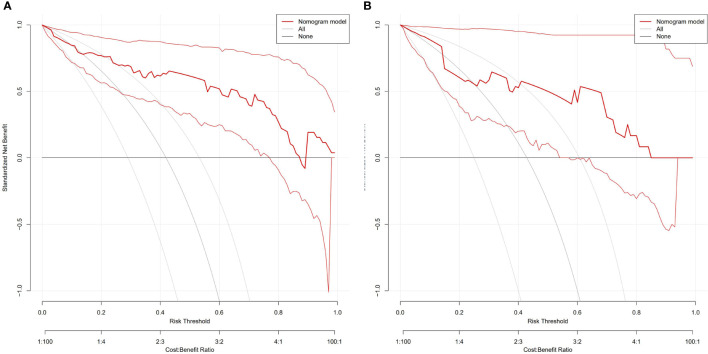
DCA of the nomogram to evaluate the clinical practicability in training **(A)** and validation cohorts **(B)**. The vertical axis displays standardized net benefit. The two horizontal axes show the correspondence between risk threshold and cost: benefit ratio. The combined nomogram achieves more net benefit across the majority of the range of threshold probabilities compared with the treat-all strategy (gray line), and treat-none strategy (horizontal black line).

**Figure 7:**
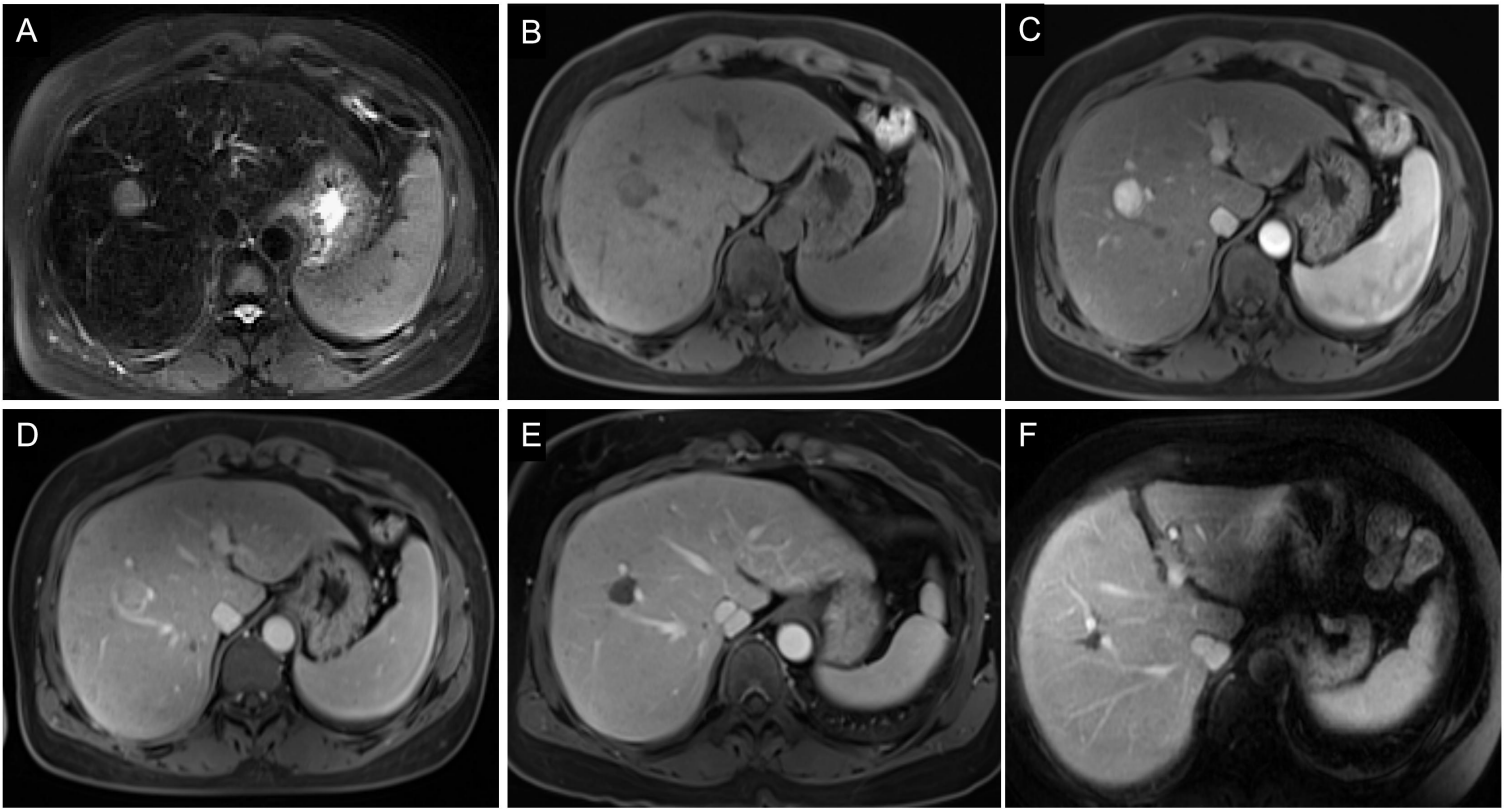
Images of a 54-year-old man with HCC without early recurrence. **(A-D)** The tumor demonstrated the typical enhancement mode of “fast in and fast out”. **(E)** There was no obvious blood supply to the tumor after RFA. **(F)** The patient was followed up for 5 years without tumor recurrence. The patient had a single tumor. The Rad-score of this patient was 1.16, and his ALB was 50.9g/l. Based on the Nomogram, his total point was about 63, indicating the risk of ER was less than 0.1.

**Figure 8:**
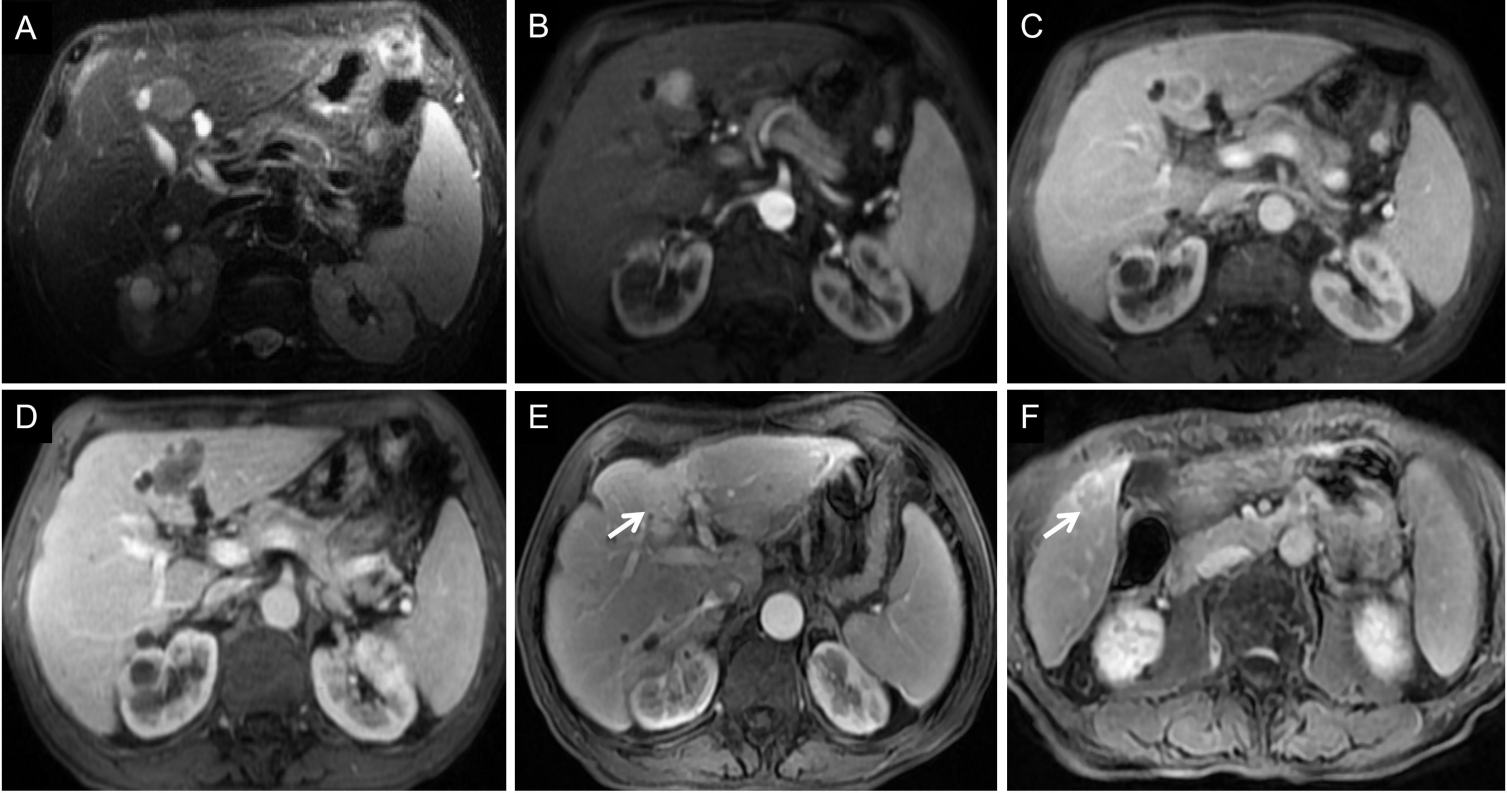
Images of a 68-year-old man with HCC with early recurrence. **(A-C)** The tumor demonstrated the typical enhancement mode of “fast in and fast out”. **(D)** No obvious blood supply was found in the tumor after RFA. **(E-F)** After follow-up for 15 months, the blood supply of the primary lesion increased, and cancerous nodules appeared in the right posterior lobe of the liver. The patient had a single tumor. The Rad-score of this patient was -1.00, and his ALB was 33g/l. According to the Nomogram, the patient’s total score was about 110, indicating that the risk of ER was between 0.8-0.9.

ALB level, tumor number, and rad-score of the mpMRI model were combined to establish a predictive nomogram model (**Figure 3**). This model showed good diagnostic efficiency in predicting the occurrence of ER, wherein the AUC in the training and validation cohorts were 0.869 and 0.812, respectively (**Figure 4**). The calibration curve showed that the predicted results for ER and non-ER were in good agreement with the actual results in the training and validation cohorts (**Figure 5**). The DCA curve showed that the nomogram model can obtain a greater net income when the threshold probability is between 20% and 90% (**Figure 6**), indicating that the nomogram can individualize the prediction of ER and has good value in clinical applications (**Figures 7**, **8**).

## 4 Discussion

Radiomics uses a variety of computer algorithms to extract massive features from medical images to improve image analysis technology and the predictive performance of imaging data (30). Hui et al. (31) analyzed the maximum cross-section of tumors on T2WI, DWI, and CE-MRI sequences in 50 patients with HCC, wherein it was found that the single radiomics features of MRI before surgical resection might predict ER. Zhao et al. (32) also found that a radiomics model based on I-T1WI, O-T1WI, T2WI, and CE-MRI sequences could effectively predict ER in patients with HCC after surgical resection. Radiomics has shown good potential for predicting postoperative recurrence of HCC. However, these studies included only patients who underwent HCC resection. Wen et al. (33) analyzed tumor radiomics features on T2WI, T1WI, and CE-MRI sequences in 111 patients with small HCC treated with surgical resection and RFA. Preoperative MRI radiomics features could predict ER with high accuracy. Shan et al. (34) further established the peritumoral feature model of CT image and found that the CT-based peritumoral radiomics can effectively predict ER of HCC. However, the subjects included not only patients who underwent RFA but also those who underwent surgical resection. Different therapeutic methods have varied effects, and their influencing factors are also different, which cannot be generalized. The ER rate of HCC with RFA is higher than that with resection. The possible reason is that the ER of HCC after RFA is related to not only tumor biology but also technology itself, such as minimal ablative margin or thermal injury-induced hepatic parenchymal hypoperfusion (35). Only patients with small HCC who were treated with RFA were included in our study. The radiomics features were obtained from the maximum cross-section of the tumor to predict the ER, and radiomics models based on single and combined sequences were established to evaluate the model with the best predictive performance.

In our study, we found that the predictive performance of the radiomics model based on the AP sequence was better than that of other models based on a single MRI sequence, which indicates that preoperative AP images are more likely to show the heterogeneity of HCC. The predictive performance of the multi-sequence combined radiomics model was better than that of the single-sequence model, and the predictive performance of the mpMRI model was the best among all radiomics models, which indicates that multi-parameter imaging contains more potential tumor heterogeneity information and can be used to analyze the prognosis of HCC after RFA. In the mpMRI radiomics model, most features were processed using local binary pattern. By comparing each voxel with its neighboring voxels and saving the results as binary numbers, it quantifies tumor heterogeneity and has a strong discrimination ability. The selected features include two first-order features (Interquartile Range, 10 Percentile), two gray-level co-occurrence matrix-based features (Cluster Shade, Difference Entropy), three gray-level run length matrix-based features (Run Entropy, Short Run Low Gray Level Emphasis), and two gray-level dependence matrix-based features (Dependence Variance, Large Dependence Low Gray Level Emphasis). These features describe the voxel intensity distribution, spatial relationship, voxel proportion, and gray relationship with neighborhood pixels in the image. The differences in tumor image texture were quantitatively evaluated from the aspects of gray-level distribution symmetry, uniformity, texture granularity, and complexity (22, 24). It has been suggested that tumor heterogeneity can affect the prognosis of patients with small HCC treated with RFA.

Because the correlation between individual radiomics features and tumor biological behavior is difficult to perceive intuitively, the rad-score calculated according to histological features and their coefficients has become a more common measurement tool (36). In this study, the mpMRI model rad-score showed good discrimination in both the training cohort and validation cohorts (AUC 0.822, 0.812, respectively). Therefore, radiomics can be used to predict the occurrence of ER in patients with small HCC who are treated with RFA. Compared to the interpretation of conventional imaging examinations, radiomics is more objective and can be used to comprehensively analyze tumors from many dimensions.

In our study, ALB levels and the number of tumors were independent risk factors for ER. Previous studies have confirmed that the number of tumors is an important prognostic factor in patients with HCC after RFA (19, 37–39). Multifocal tumors were closely related to small HCC, suggesting that tumors are more invasive, more prone to occult metastasis, and have a poor prognosis, similar to previous studies, which have shown that ALB level is also a risk factor for HCC recurrence and prognosis (19, 37, 40). Patients with low ALB levels were more likely to relapse soon after RFA. This may be because ALB reflects the synthetic and reserve function of the liver, wherein the recurrence of HCC is accompanied by impairment of normal hepatocyte function, and the continuous decline of synthetic ALB. Although this study concluded that ALB is an independent risk factor for ER after RFA for small HCC, further Child-Pugh classification did not show any correlation with ER. A possible reason is that this study was a retrospective study, wherein the subjects were patients with very early or early HCC, and the number of patients with poor liver function grade was lower.

Finally, a nomogram model including clinical features and rad-score was established to predict the ER of small HCC after RFA, and its diagnostic efficiency was high, indicating that the degree of discrimination of the nomogram model was good. Compared to the individual image group model, the overall prediction performance of the combined model was better than that of the single radiomics model. Considering that the combined model covers more aspects, its performance is more stable. The calibration curve showed good consistency between the predicted and actual results of the ER and non-ER groups in the training and validation cohorts. Therefore, the nomogram model has good differentiation and calibration, making it convenient for clinical guidance in performing personalized follow-up to provide timely intervention for patients at high risk of ER. For patients with high risk of ER, expanding the scope of RFA or trying new adjuvant therapy may reduce the incidence of ER (19, 41). In a previous study, Laimer et al. showed that the minimal ablative margin was the only significant independent predictor of local tumor progression, and the relative risk decreased by 30% for every 1 mm increase in minimal ablative margin (41). Active follow-up should be undertaken after RFA to detect tumor recurrence as early as possible.

### 4.1 Limitations

First, as this was a single-center retrospective study, it may be difficult to avoid selection bias, and the sample size was limited. More accurate conclusions need to be drawn after further evaluation by means of a joint multicenter study and by expanding the sample size. Secondly, 1.5T and 3.0T scanners with different parameters were used for MRI inspection, which is not conducive to the normalization of signal strength. However, before image feature extraction, we preprocessed the image to reduce possible variability. Third, taking the maximum cross-section of the tumor as the ROI may not be the best way to represent the tumor. However, a 2D analysis can be considered a simplification. Previous studies have shown that sufficient data can be obtained on the maximum cross-section of the tumor, and 2D analysis is not as cumbersome and time-consuming as 3D analysis. Finally, the image group analysis of this study was carried out using the AK software, which is integrated with a variety of algorithm components and has the advantages of easy operation. However, the process of data processing is carried out in the background, and the underlying data of some processes cannot be obtained. It was necessary to utilize the R software to further verify and expand the research results.

### 4.2 Conclusion

Radiomics has the potential to predict ER after RFA of small HCC. The combined model based on mpMRI and clinical features shows good differentiation, calibration, and clinical practical value. It can be used to identify patients at a high risk of ER after RFA for small HCC, which is helpful for personalized risk stratification and further treatment decision-making in patients with small HCC.

## Data availability statement

The raw data supporting the conclusions of this article will be made available by the authors, without undue reservation.

## Ethics statement

The studies involving human participants were reviewed and approved by Institutional Review Board of the 900th Hospital of the Joint Logistics Support Force. Written informed consent for participation was not required for this study in accordance with the national legislation and the institutional requirements.

## Author contributions

Conceptualization: QZ and XZ; methodology: XZ, CW, and YL; formal analysis: XZ, CW, DZ, YL, XW, ZH, and QZ; resources: YL, XW, ZH, and QZ; writing—original draft preparation: XZ; writing—review and editing: XZ and QZ; funding acquisition: QZ. All authors contributed to the article and approved the submitted version.

## Acknowledgments

We thank the radiographers at the 900th Hospital of the Joint Logistics Support Force for scanning the patients and data collections in this study.

## Conflict of interest

YL was employed by GE Healthcare.

The remaining authors declare that the research was conducted in the absence of any commercial or financial relationships that could be construed as a potential conflict of interest.

## Publisher’s note

All claims expressed in this article are solely those of the authors and do not necessarily represent those of their affiliated organizations, or those of the publisher, the editors and the reviewers. Any product that may be evaluated in this article, or claim that may be made by its manufacturer, is not guaranteed or endorsed by the publisher.

## Abbreviations

AFP, alpha-fetoprotein; ALB, albumin; AP, arterial phase; AUC, area under the receiver operating characteristic curve; BCLC, Barcelona Clinical Liver Cancer; CE-MRI, contrast-enhanced MRI; CI, confidence interval; DCA, decision curve analysis; DP, delayed phase; DWI, diffusion-weighted imaging; ER, early recurrence; HCC, hepatocellular carcinoma; HR, hazard ratio; ICC, intra-group correlation coefficient; mpMRI, multi-parametric MRI; MRI, magnetic resonance imaging; PVP, portal venous phase; rad-score, radiomics score; RFA, radiofrequency ablation; ROC, receiver operating characteristic; ROI, regions of interest.
